# P01-012 – Evaluation of autonomic function in FMF

**DOI:** 10.1186/1546-0096-11-S1-A16

**Published:** 2013-11-08

**Authors:** N Aktay Ayaz, N Melikoğlu Kiplapinar, G Keskindemirci, G Aydogan, O Isal Tosun, A Guzeltas, E Odemis

**Affiliations:** 1 Pediatric Rheumatology, Istanbul Kanuni Sultan Suleyman Education and Research Hospital, Turkey; 2 Pediatric Cardiology, Istanbul Mehmet Akif Ersoy Thoracic and Cardiovascular Surgery Center, Istanbul, Turkey

## Introduction

Familial Mediterranean fever (FMF) is characterized by acute and recurrent attacks of fever and polyserositis. It is associated with conduction disturbances and rhythm abnormalities. Heart rate variability (HRV) is the term used to indicate the fluctuation in cardiac frequency over time. HRV analysis is used to evaluate the condition of the autonomous nervous system, which regulates general cardiac condition and cardiac activity. Significant correlations between cardiovascular mortality and the autonomous nervous system have been evidenced in the last twenty years. In adult FMF patients abnormal heart rate variability (HRV) parameters were found, suggesting to autonomic dysfunction.

## Objectives

To assess cardiac autonomic functions in FMF patients during childhood period.

## Methods

A prospective randomized clinical trial was performed by a tertiary referral pediatric cardiology and a pediatric rheumatology center. The study group consisted of 53 patients with FMF (28 female, 25 male) that were followed-up by the pediatric rheumatology out-patient clinic. They were all under colchicine treatment. The control group was chosen from age and sex matched 44 healthy children (21 female, 23 male). All participants underwent 24-hour Holter rhythm monitoring (CardioNavigator Plus Impresario Medical Spider view, 3.07.0158, Delmar Reynolds; Paris, France). The HRV parameters were evaluated in both groups.

## Results

The mean age of the study group was 11.6±3.5 years and the control group was 10.4±3.4 years. Height and weight of the study group were 143.2±19 cm and 37.9± 11.7 kg respectively. The control group’s height and weight were 143.6±18.1 cm and 38.5±14.1 kg respectively. The mean duration of colchicine treatment was 43.4±41.5 months. The time-domain analysis of HRV revealed similar values of mean “standard deviation of all NN intervals” (SDNN;152.3±46.2 vs 143.13±41.99 msec, p=0.423), “SD of the 5 min mean RR intervals” (SDANN;131.3±36.3 vs 128.6±36.5 msec, p=0.451 ), “root square of successive differences in RR interval” (RMSSD; 70.8±53.5 vs 69±33.6 msec, p=0.481), and “proportion of differences in successive NN intervals >50 ms” (PNN50; 21.2±14 vs 21.3±12.1%, p=0.524), “triangular interpolation of NN interval histogram” (TINN; 623±219 vs 615±170 msec, p=0.451) and “HRV index” (20.8±6.8 vs 20.3±5.2 msec, p=0.633) in both groups. Frequency domain analysis revealed similar values of high frequency (HF; 48.2±13.9 vs 46.3±14.8, p=0.451), low frequency (LF; 42.5±12.7 vs 44±15.3, p=0.451) and LF/HF (1.08±0.84 vs 1.31±1.5, p=0.542) components in both groups.

## Conclusion

Autonomic nervous system has an important role in the supervision of cardiac functions. In adult patients with uncomplicated FMF there are two published studies about the autonomic dysfunction, one revealing autonomic indices abnormalities and the other with similar normal autonomic function compared to healthy subjects. As being the first study concerning the autonomic function in children with FMF, we had found no significant differrences between both groups. This may be atrributed to the shorter duration and uncomplicated course of disease in children with FMF.

## Disclosure of interest

None declared

